# The efficacy and safety of modified FOLFIRINOX as first-line chemotherapy for Chinese patients with metastatic pancreatic cancer

**DOI:** 10.1186/s40880-019-0367-7

**Published:** 2019-05-08

**Authors:** Zhi-Qiang Wang, Fei Zhang, Ting Deng, Le Zhang, Fen Feng, Feng-Hua Wang, Wei Wang, De-Shen Wang, Hui-Yan Luo, Rui-Hua Xu, Yi Ba, Yu-Hong Li

**Affiliations:** 1 Department of Medical Oncology, State Key Laboratory of Oncology in South China, Collaborative Innovation Center for Cancer Medicine, Sun Yat-sen University Cancer Center, 651 Dongfeng East Road, Guangzhou, 510060 Guangdong People’s Republic of China; 2 Department of Gastrointestinal Medical Oncology, National Clinical Research Center of Cancer, Key Laboratory of Cancer Prevention and Therapy, Tianjin Medical University Cancer Institute and Hospital, Tianjin, 300060 People’s Republic of China; 3 Cancer Center, The First People’s Hospital of Foshan, Foshan, 528000 Guangdong People’s Republic of China

**Keywords:** Pancreatic cancer, Metastatic, Chemotherapy, FOLFIRINOX, Dose modification, First-line, Multicenter, Overall response rate, Efficacy, Safety

## Abstract

**Background:**

Oxaliplatin, irinotecan, 5-fluorouracil, and l-leucovorin (FOLFIRINOX) has become one of the first-line treatment options for advanced pancreatic cancer (PC). However, the relatively high rate of grade 3 or 4 adverse events associated with the standard dosage of FOLFIRINOX limits its widespread use in clinical practice. In this study, we were to evaluate the efficacy and safety of a modified FOLFIRINOX regimen as a first-line chemotherapy for Chinese patients with metastatic PC.

**Methods:**

Patients with histologically confirmed primary metastatic pancreatic adenocarcinoma with an Eastern Cooperative Oncology Group (ECOG) performance status score of 0–2 were recruited to receive the modified FOLFIRINOX regimen (intravenous infusion of oxaliplatin, 65 mg/m^2^; irinotecan, 150 mg/m^2^; l-leucovorin, 200 mg/m^2^; and 5-fluorouracil, 2400 mg/m^2^, repeated every 2 weeks). The treatment was continued for 12 cycles unless the patient had progressive disease (PD), stable disease (SD) with symptom deterioration, unacceptable adverse events, or requested to terminate the treatment prematurely. The primary endpoint was objective response rate (ORR).

**Results:**

Sixty-five patients were enrolled from July 2012 to April 2017 in three institutions, and they all received at least one cycle of chemotherapy, with a median of 8 cycles (range 1–12 cycles). No complete response was observed. Twenty-one (32.3%) patients had partial responses, and 27 (41.5%) had SD. The ORR and disease control rate of the study cohort was 32.3% and 73.8%. The estimated median overall survival and progression-free survival were 11.60 (95% confidence interval [CI] 8.76–14.44) and 5.77 (95% CI 5.00–6.54) months. Major grade 3 or 4 adverse events included neutropenia (12.3%) and diarrhea (6.2%). No treatment-related death was observed.

**Conclusions:**

Modified FOLFIRINOX was well-tolerated and might be a promising option as first-line therapy for Chinese patients with metastatic PC.

*Trial registration* ClinicalTrials.gov, NCT02028806. Registered 7 January 2014, https://clinicaltrials.gov/ct2/show/NCT02028806

## Background

Pancreatic cancer (PC) is the fourth leading cause of cancer-related death worldwide [1]. Despite decades of efforts, it is still one of the deadliest solid malignancies, with a 5-year survival rate of less than 5% [2]. Gemcitabine has become the standard care for patients with locally advanced or metastatic PC since 1997 [3]. The addition of albumin-bound paclitaxel (nab-paclitaxel) to gemcitabine has significantly improved the patient’s survival rate and has become the newly accepted standard of treatment [4]. However, the combinations of gemcitabine with other cytotoxic or molecularly targeted agents have generally shown no substantial clinical improvement when compared with gemcitabine alone [5–7].

The PRODIGE 4/ACCORD 11 study [5] exploring FOLFIRINOX (a combination chemotherapy regimen of oxaliplatin, irinotecan, 5-fluorouracil, and l-leucovorin) as a first-line treatment in patients with advanced PC was performed by a French consortium study group. When comparing the FOLFIRINOX regimen with single-agent gemcitabine, they found that the FOLFIRINOX regimen was associated with significantly prolonged overall survival (OS, 11.1 months vs 6.8 months, *P *< 0.001) and progression-free survival (PFS, 6.4 months vs 3.3 months, *P *< 0.001). Moreover, the objective response rate (ORR) of patients in the FOLFIRINOX group was higher than that of patients in the gemcitabine group (31.6% vs 9.4%, *P *< 0.001). Furthermore, the observed definitive degradation of the quality of life was significantly lower in the FOLFIRINOX group (31% vs 66%, *P* < 0.001). Thus, FOLFIRINOX became one of the standard options for patients with advanced PC and has demonstrated good performance status in North America and Europe. However, grade 3 or 4 adverse events (45.7% grade 3 or 4 neutropenia, 5.4% febrile neutropenia, 12.7% diarrhea, 9.1% thrombocytopenia, 9.0% sensory neuropathy) were frequently observed in the FOLFIRINOX group even when using the median relative dose intensity of 5-fluorouracil, irinotecan, and oxaliplatin which 82%, 81%, and 78% of the initial dose, respectively [5]. As a result, many dose modifications have been reported and have demonstrated reduced adverse events without compromising the treatment efficacy in both metastatic and adjuvant settings [8–13].

However, data on the efficacy and safety of FOLFIRINOX in Chinese patients with metastatic PC is still limited. Therefore, in this study, we aimed to evaluate the efficacy and safety of a modified FOLFIRINOX as a first-line chemotherapy regimen for Chinese patients with metastatic PC.

## Patients and methods

### Patient population

Patients who were radiologically and histologically diagnosed with primary metastatic pancreatic adenocarcinoma between July 2012 and April 2017 were enrolled in this multicenter, single-arm, prospective phase II study. Inclusion criteria included an age of 18 years or older and an Eastern Cooperative Oncology Group (ECOG) performance status score of 0, 1 or 2, with at least one measurable metastatic lesion and adequate hematological, liver, and renal functions (hemoglobin ≥ 90 g/L, neutrophil count ≥ 2.0 × 10^9^/L, platelet count ≥ 90 × 10^9^/L, total bilirubin ≤ 1.2 × upper limit of normal, aspartate transaminase and alanine transaminase ≤ 2.5 × upper limit of normal, and creatinine ≤ 1.25 × upper limit of normal). Prior adjuvant therapy (including adjuvant radiotherapy or chemotherapy > 4 weeks) was allowed. Patients with postoperative recurrence were also enrolled.

Exclusion criteria were an age of 76 years or older, pancreatic malignancies other than adenocarcinoma, a history of other types of cancer, active and uncontrolled medical diseases such as severe sepsis and septic shock, symptomatic peripheral neuropathy > grade 2, a history of palliative radiotherapy/immunotherapy, and pregnancy or breastfeeding women. Written informed consent was obtained from all patients. The study was approved by the independent ethics committees of Sun Yat-sen University Cancer Center, Tianjin Medical University Cancer Institute and Hospital, and The First People’s Hospital of Foshan and was performed in accordance with the Declaration of Helsinki.

### Treatment and assessment

Patients were given a 30-min intravenous infusion of antiemetic and then treated with modified FOLFIRINOX (a 2-h intravenous infusion of oxaliplatin at 65 mg/m^2^, immediately followed by a 2-h intravenous infusion of l-leucovorin at 200 mg/m^2^, a 1.5-h intravenous infusion of irinotecan at 150 mg/m^2^, and a 46-h continuous infusion of fluorouracil at 2400 mg/m^2^, repeated every 2 weeks). Granulocyte colony-stimulating factor was allowed for those who suffered grade 3 or 4 neutropenia. The treatment was continued for 12 cycles unless the patient had progressive disease (PD), stable disease (SD) with symptom deterioration, unacceptable adverse events, or requested to terminate the treatment prematurely.

### Study design

The sample size was estimated by the Simon’s two-stage design. The expected ORR for the modified FOLFIRINOX was initially assumed to be 30%. If the ORR was less than 10%, the modified FOLFIRINOX was considered to be ineffective. Due to the possible occurrence of the first type of error (α = 0.05) and the possible occurrence of the second type of error (β = 0.10), 18 patients were needed in the first stage of the study. If more than 2 patients were responsive to the treatment, the second stage would be initiated, and 17 more patients would be enrolled. Considering a withdrawal rate of 15%, a total of 40 patients were needed in this study.

### Evaluation of response

Patients completing at least one cycle of the modified FOLFIRINOX with at least one follow-up tumor assessment were considered evaluable for response. Computed tomography (CT) of the chest, abdomen, and pelvic cavity was repeated every 4 cycles. The treatment response was determined in accordance with the Response Evaluation Criteria in Solid Tumors (RECIST, version 1.0) by the investigators. Adverse events were assessed according to the Common Terminology Criteria for Adverse Events (CTCAE, version 4.0) on days 1, 8, 15 of the treatment cycle. Second-line treatment was strongly recommended for those who were still with good performance status after PD was observed during the treatment period.

### Study endpoints

The primary endpoint of this phase II trial was ORR, and the secondary endpoints were disease control rate (DCR), OS, PFS and the rates of adverse events. ORR was determined as the percentage of patients who had a complete (CR) or partial response (PR). DCR was defined as the percentage of patients who had a CR, PR, or SD. In addition, OS was calculated from the date of enrollment to death from any cause. PFS was defined as the time interval from recruitment until radiological or clinical progression or death from any cause.

### Follow-up

All patients received regular blood tests including tumor markers detection and CT scans of the chest, abdomen, and pelvic cavity every 2 months unless they had PD. Patients with PD after modified FOLFIRINOX treatment were followed-up every 3 months by telephone until death. The end of follow-up period was December 20, 2018.

### Statistical analysis

Descriptive statistical analysis was used to determine the ORR and DCR. The OS and PFS were estimated using the Kaplan–Meier method. Log-rank test was used for statistical comparisons between survival curves. All statistical analyses were carried out using the SPSS software, version 22.0 (SPSS Inc., Chicago, IL, USA). A two-sided *P* value of less than 0.05 was considered significant.

## Results

### Patient characteristics

Between July 2012 and April 2017, a total of 65 patients with histologically confirmed primary metastatic PC from three centers (Sun Yat-sen University Cancer Center [*n* = 38], Tianjin Medical University Cancer Institute and Hospital [*n* = 21], and The First People’s Hospital of Foshan [*n* = 6]) were included in this study. There were 43 men (66.2%) and 22 women (33.8%) with a median age of 56 years (range 23–74 years). Thirty-two (49.2%) patients had tumors located in the head of the pancreas, while there were 13 (20.0%) and 6 (9.2%) patients with tumors located in the body and tail of the pancreas, respectively. Fourteen (21.6%) patients had multicentric PC. Six patients had an ECOG performance score of 2, and the remaining 59 patients had a score of 0 or 1. The baseline clinicopathologic characteristics are summarized in Table 1.

**Table 1:** Demographic and baseline characteristics of the enrolled patients

Clinicopathological	No. of patients
Characteristics	*n*, (%)
Gender	
Male	43 (66.2)
Female	22 (33.8)
ECOG performance status score	
0	21 (32.3)
1	38 (58.5)
2	6 (9.2)
Primary tumor location	
Head	32 (49.2)
Body	13 (20.0)
Tail	6 (9.2)
Multicentric	14 (21.6)
No. of metastatic sites involved	
Median	1
Range	1–4
Metastatic tumor sites	
Liver	53 (81.5)
Lung	14 (21.5)
Lymph nodes	19 (29.2)
Peritoneum	15 (23.1)
Others	7 (10.8)
Cycles of mFOLFIRINOX	
Median	8
Range	1–12

*ECOG* Eastern Cooperative Oncology Group, *mFOLFIRINOX* modified 5-fluorouracil, irinotecan, leucovorin, and oxaliplatin

### Responses

A total of 469 cycles of chemotherapy were delivered, with a median of 8 cycles per patient (range 1–12 cycles). The responses to therapy are summarized in Table 2. Of the 65 patients, 21 (32.3%) achieved PR, and 27 (41.5%) had SD; no CR was observed, leading to an ORR and DCR of 32.3% (21/65) and 73.8% (48/65). One case could not be evaluated due to the lack of CT scan after receiving 3 cycles of treatment. No CR or PR was observed in patients with an ECOG performance score of 2.

**Table 2:** Objective responses and survival of the study population

Variable	No. of Patients [cases (%)]	Median PFS	Median OS
		(95% CI, months)	(95% CI, months)
Best response			
Complete response	0	**–**	–
Partial response	21 (32.3)	7.80 (4.86–10.74)	18.17 (12.89–23.45)
Stable disease	27 (41.5)	7.00 (4.43–9.57)	13.00 (9.68–16.32)
Progressive disease	16 (24.6)	1.93 (1.59–2.28)	4.30 (3.58–5.02)
Could not be evaluated	1 (1.5)	–	–
Objective response rate^a^	21 (32.3)	–	–
Disease control rate^b^	48 (73.8)	–	–

*OS* overall survival, *PFS* progression free survival, *CI* confidence interval

^a^The rate of objective response was defined as the percentage of patients who had a complete response or a partial response

^b^The rate of disease control was defined as the percentage of patients who had a complete response, partial response, or stable disease

### Survival

**Fig. 1:**
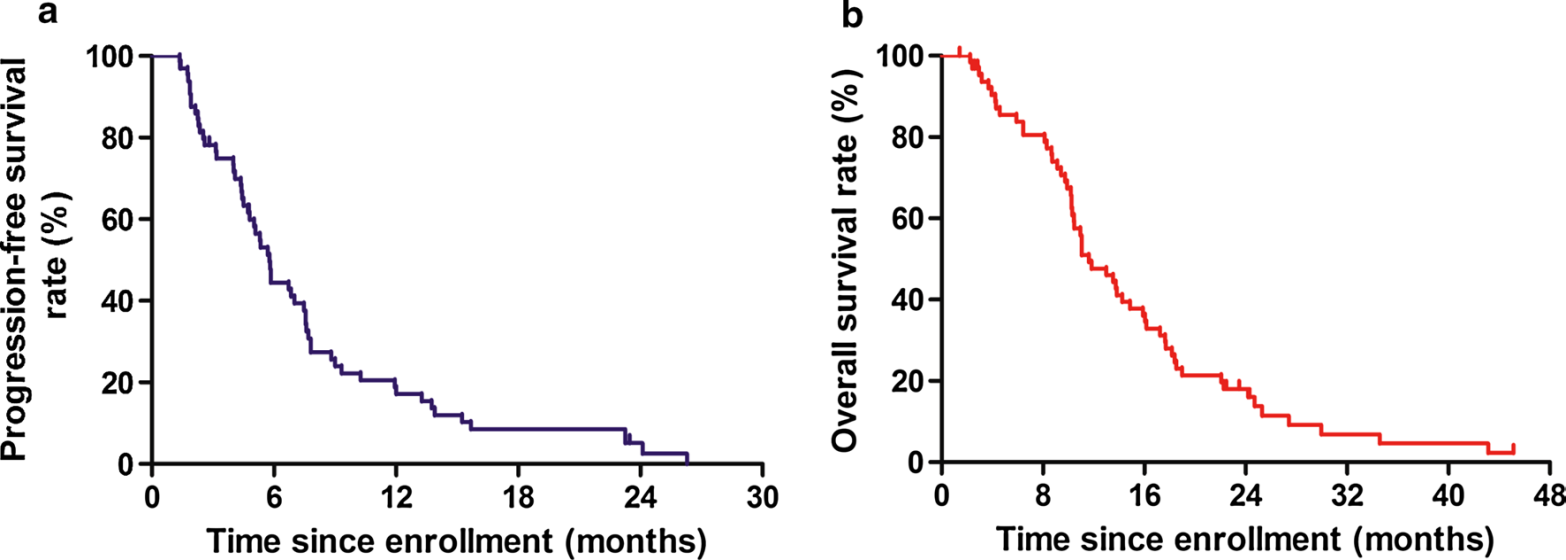
Kaplan-Meier survival curves of **a** progression-free survival (PFS) and **b** overall survival (OS) of the 65 enrolled patients

The median follow-up time was 16.70 (range 1.43–45.13) months. At the end of the study, 59 (90.8%) of the 65 patients had PD, and 57 (87.7%) died. The estimated median PFS and OS of the entire study cohort were 5.77 (95% confidence interval [CI] 5.00–6.54) and 11.60 (95% CI 8.76–14.44) months (Fig. 1).

**Fig. 2:**
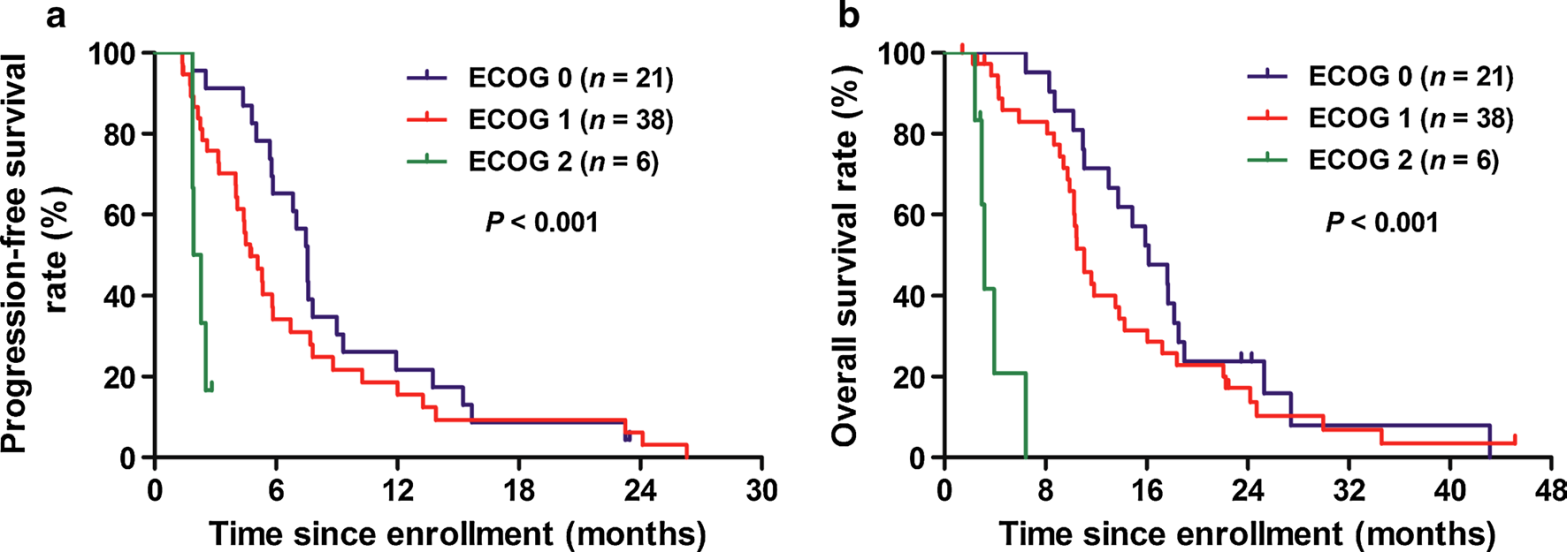
Kaplan-Meier survival curves of **a** progression-free survival (PFS) and **b** overall survival (OS) of the 65 enrolled patients stratified by the Eastern Cooperative Oncology Group (ECOG) performance status scores (log-rank test of ECOG 0 vs 1 vs 2)

In addition, the estimated median PFS for patients with an ECOG performance score of 0, 1, and 2 were 7.57 (95% CI 7.37–7.77), 4.73 (95% CI 3.55–5.92) and 1.93 (95% CI 1.41–2.45) months, respectively (ECOG 0 vs 1 vs 2: *P* < 0.001; Fig. 2a), and the estimated median OS were 16.17 (95% CI 12.03–20.30), 11.07 (95% CI 9.57–12.57) and 3.17 (95% CI 2.68–3.65) months, respectively (ECOG 0 vs 1 vs 2: *P* < 0.001; Fig. 2b).

### Safety

The common treatment-related hematologic and nonhematologic adverse events are summarized in Table 3. The most common adverse events were neutropenia, anemia, loss of appetite, nausea, vomiting, fatigue, and alanine aminotransferase elevation. Major grade 3 or 4 adverse events included neutropenia in 8 (12.3%) patients and diarrhea in 4 (6.2%) patients. Eight patients needed dose reduction due to severe diarrhea (*n* = 4) or neutropenia (*n* = 4). 5-Fluorouracil and/or irinotecan infusion were the most commonly adjusted agents. Of the 6 patients with an ECOG performance score of 2, only one developed grade 4 neutropenia and recovered after 1 week of hospitalized treatment. No treatment interruption or treatment-related death occurred.

**Table 3:** The rate of treatment-related adverse events of the study cohort

Adverse event	Frequency [cases (%)]			
	Grade 1	Grade 2	Grade 3	Grade 4
Hematologic				
Neutropenia	20 (30.8)	15 (23.1)	4 (6.2)	4 (6.2)
Thrombocytopenia	9 (13.8)	2 (3.1)	0	0
Anemia	24 (36.9)	3 (4.6)	0	1 (1.5)
Nonhematologic				
Loss of appetite	34 (52.3)	18 (27.7)	0	0
Nausea	26 (40.0)	18 (27.7)	0	0
Vomiting	14 (21.5)	19 (29.2)	1 (1.5)	0
Mucosal inflammation	14 (21.5)	1 (1.5)	0	0
Diarrhea	20 (30.8)	4 (6.2)	4 (6.2)	0
Alopecia	52 (80.0)	13 (20.0)	–	–
Fatigue	25 (38.5)	10 (15.4)	0	0
Sensory neuropathy	18 (27.7)	3 (4.6)	0	0
Weight loss	7 (10.8)	0	0	0
Constipation	7 (10.8)	1 (1.5)	0	0
Alanine aminotransferase elevation	22 (33.8)	7 (10.8)	1 (1.5)	0
Aspartate aminotransferase elevation	12 (18.5)	4 (6.2)	0	0
Bilirubin elevation	4 (6.2)	0	0	0
Allergic reaction	1 (1.5)	0	0	0
Rash	1 (1.5)	0	0	0
Hand-foot syndrome	1 (1.5)	0	0	0
Singultus	1 (1.5)	0	0	0

### Second-line therapy

Second-line therapy was recommended for patients with good performance status and adequate organ functions who had PD after the modified FOLFIRINOX chemotherapy (*n* = 20). Gemcitabine plus nab-paclitaxel was the most commonly used second-line regimen and was prescribed in 14 patients. Other patients received gemcitabine (*n* = 3) or S1 (*n* = 3) monotherapy. No patients underwent third-line therapy due to a decrease in ECOG performance status after the second-line chemotherapy.

## Discussion

In the present study, in an attempt to reduce the risk of adverse events of the FOLFIRINOX regimen, dose modification was performed by using 65 mg/m^2^ and 150 mg/m^2^ of oxaliplatin and irinotecan based on their median relative dose intensities of their initial prescribed dosage used in the PRODIGE 4/ACCORD 11 study [5], and removed the intravenous bolus of 5-fluorouracil due to its notable myelosuppression. Based on such modifications, we found that our modified FOLFIRINOX regimen demonstrated encouraging anti-cancer activity in Chinese patients with metastatic PC. The ORR and DCR were reported to be 32.3% and 73.8%. Moreover, the estimated median OS and PFS were 11.60 and 5.77 months, which were consistent with those treated with the full-dose FOLFIRINOX [5].

Other reported modified FOLFIRINOX regimens were usually established by removing the intravenous bolus of fluorouracil and reducing the oxaliplatin and irinotecan dosages to 78%–88% and 64%–81% of the full dose (Table 4) [11, 14, 15]. Stein et al. [14] reported the efficacy of a modified FOLFIRINOX (the doses of irinotecan and bolus 5-fluorouracil reduced by 25%) with an ORR of 35.1% and median OS and PFS of 10.2 and 6.1 months. Meanwhile, Li et al. [15] evaluated the efficacy and safety of a modified FOLFIRINOX (the doses of oxaliplatin and irinotecan adjusted to 85% and 75% of the full doses, without intravenous bolus of 5-fluorouracil) in 62 metastatic PC patients treated at a single institution. They found that a DCR of 60% was achieved of which 13 patients had CR or PR, leading to an ORR of 32.5%, and the median OS and PFS were 10.3 and 7.0 months. Thus, combined with the results from these previous studies, we strongly suggest that a modified FOLFIRINOX regimen can have comparable treatment efficacy as to that of the original regimens reported in the PRODIGE 4/ACCORD 11 study [5].

**Table 4:** The efficacy and safety of standard and modified FOLFIRINOX regimen in advanced pancreatic cancer patients

Events	Standard regimen	Modified regimen			
	Conroy et al. [5]	Yoshida et al. [11]	Stein et al. [14]	Li et al. [15]	Present study
Patients (*n*)	171	31	68	62	65
Disease (*n*)					
Locally advanced	0	0	31	0	0
Metastatic	171	31	37	62	65
ECOG performance score (*n*)					
0	64	25	17	38	21
1	106	6	20	24	38
2	1	0	0	0	6
Dose (mg/m^2^)					
Oxaliplatin	85	85	85	68	65
Irinotecan	180	150	135	135	150
5-Fluorouracil	2400	2400	2400	2400	2400
Survival (months)					
Median OS	11.1	14.9	10.2	10.3	10.9
Median PFS	6.4	7	6.1	7	6.7
Response (*n*)					
Complete response	1	0	0	0	0
Partial response	53	12	13	13	21
Stable disease	66	11	19	11	27
Progressive disease	26	8	5	16	16
Objective response rate (%)	31.6	38.7	35.1	32.5	32.3
Disease control rate (%)	70.2	74.2	86.5	60	73.8
Adverse event (%)					
Neutropenia (grade 3/4)	45.7	83.9	12.2	29	12.4
Fatigue (grade 3/4)	23.6	NE	12.2	0	0
Diarrhea (grade 3/4)	12.7	6.5	16.2	0	6.2
Vomiting (grade 3/4)	14.5	3.2	2.7	0	1.5
Sensory neuropathy (grade 3/4)	9	9.7	2.7	0	0
Sensory neuropathy (grade 1/2)	NE	58.1	NE	0	32.3

*NE* not evaluated

In regard to the therapeutic safety, our results showed that this modified FOLFIRINOX regimen was well-tolerated, had a relatively low number of adverse events (grade 3 or 4 neutropenia 13.4%, no febrile neutropenia; grade 3 diarrhea 6.2%, grade 2 sensory neuropathy 4.2%), without treatment-related death. In comparison with other modified FOLFIRINOX regimens [11, 14], the rates of adverse events were different in Chinese PC patients. The rate of grade 3 diarrhea in this study was significantly higher than that reported by Li et al. [15], which might be attributed to the relatively higher dose of irinotecan in the present study (150 mg/m^2^ vs 135 mg/m^2^) (Table 4). Moreover, the median number of cycles of modified FOLFIRINOX delivered per patient in this study were 8 (oxaliplatin with a cumulative dosage of 520 mg/m^2^), which could explain why there were more patients suffering from grade 1 or 2 sensory neuropathy (21 cases, 32.3%) compared to those in Li et al.’s study [15].

Furthermore, in the present study, patients were included regardless of their ECOG performance scores. Among them, 6 patients had an ECOG performance status score of 2 and showed good tolerance of treatment with the modified FOLFIRINOX regimen, except for one patient who developed grade 4 neutropenia but recovered after 1 week of hospitalized treatment. In addition, the estimated median PFS and OS were 1.93 (95% CI 1.41–2.45) months and 3.17 (95% CI 2.68–3.65) months. This preliminary result showed that although the adverse events of modified FOLFIRINOX in ECOG performance status 2 patients were manageable, their PFS and OS were short, which might be attributed to the unwillingness of the patients to receive subsequent treatment. For these patients, further studies might be needed to compare the efficacy and safety of the modified FOLFIRINOX with the standard monotherapy regimen.

There are some limitations of the present study that needs to be addressed. First, although the patients were recruited from three hospitals, only 65 patients were enrolled and the study period was relatively long, nearly 5 years. Second, the small sample size may have partly affected the observed results of this study. Third, we could not evaluate the patients’ quality of life due to insufficient data. Therefore, we suggest that further studies on a larger cohort of patients are needed to confirm the efficacy and safety of this regimen.

## Conclusions

The efficacy of this modified FOLFIRINOX was comparable to that of the original regimen but with a notably lower rate of adverse events. This modified FOLFIRINOX regimen might be a promising first-line chemotherapy option for Chinese patients with metastatic PC.

## Abbreviations

FOLFIRINOX: oxaliplatin, irinotecan, fluorouracil and l-leucovorin
PC: pancreatic cancer
ECOG: Eastern cooperative oncology group
ORR: objective response rate
CI: confidence interval
OS: overall survival
PFS: progression-free survival
PD: progressive disease
SD: stable disease
RECIST: response evaluation criteria in solid tumors
CT: computed tomographic
CTCAE: common terminology criteria for adverse events
DCR: disease control rate
PR: partial response
CR: complete response
